# The Theory and Practice of Sensory Evaluation of Vinegar: A Case of Italian Traditional Balsamic Vinegar

**DOI:** 10.3390/foods14050893

**Published:** 2025-03-05

**Authors:** Fusheng Chen, Yanqin Ma, Giuseppe Corradini, Paolo Giudici

**Affiliations:** 1 School of Life Sciences, Guizhou Normal University, Guiyang 550025, China; 2 College of Food Science and Technology, Huazhong Agricultural University, Wuhan 430070, China; mayq@webmail.hzau.edu.cn; 3 State Key Laboratory of Agricultural Microbiology, Huazhong Agricultural University, Wuhan 430070, China; 4 Hubei International Scientific and Technological Cooperation Base of Traditional Fermented Foods, Huazhong Agricultural University, Wuhan 430070, China; 5 Liceo Corso, 42015 Correggio, Italy; giuseppecorradini@libero.it; 6 Società Agraria di Reggio Emilia, 42100 Reggio Emilia, Italy

**Keywords:** sensory analysis, vinegar, Italian Traditional Balsamic Vinegar

## Abstract

Sensory analysis is a very powerful and useful tool that is used for a variety of foods. But for vinegar, the relevant sensory evaluation system is not satisfactory since there are still some issues, such as the tendency for score conservatism, descriptor redundancy, sensory fatigue and other cognitive issues. In this review, the theory of the sensory evaluation of vinegar is first introduced, and then the application of sensory evaluation is summarized for vinegar, especially for Italian Traditional Balsamic Vinegar. By improving the scoring system and enhancing the tasting conditions, the reliability of vinegar sensory evaluation can be further increased and provide a solid support for vinegar quality control and market promotion.

## 1. Introduction

Sensory analysis, which emerged in the 1940s and has developed significantly in subsequent decades, is very important for foods, and in recent years, it has been widely used in the fields of food quality control, product development and market research [1,2,3]. It is a scientific specialty used to evaluate, study, and interpret the responses of group members to food properties by the senses of vision, smell, taste, touch and hearing [4]. Sensory analysis is applied to determine the specificity or typicality of a food, especially for dairy products [5,6], wine [7] and distilled liquor [8]. For instance, sensory analysis is particularly commonly used in wines, the sensory properties of which, such as aroma complexity, taste balance and aftertaste expression, have long been considered as a central value of the product, and relevant sensory analysis tools and standardized methods are relatively developed [9,10]. For the sensory evaluation of wines, a clear system of descriptive vocabularies, e.g., body, acidity and tannin, has been established [11,12]. Paissoni et al., 2023 [11], tracked changes in sensory properties caused by different winemaking regimens and examined the overall quality of different wines. Vinegar, an acidic condiment beloved worldwide, holds a significant position in the culinary and cultural traditions of many countries and regions in the world [13]. Although sensory evaluation has been extensively used in assessing the quality of various fermented foods [14], its application in assessing vinegar started relatively late, resulting in limited accumulated experience and expertise [15]. This situation highlights a need for advancing the theory and methodology of sensory analysis for vinegar, particularly for traditional vinegar. Previous reports have shown that sensory analysis has been employed to distinguish vinegars based on their raw materials [16] and production methods [17]. However, these vinegar sensory evaluations inherently involve many elements, such as culture, education, environment, habits, sensory abilities and personal preferences, leading to considerable variability in data from the tasters and impacting evaluation outcomes of vinegar [13,18,19,20]. Therefore, understanding and minimizing these biases is essential for improving the scientific rigour and reliability of vinegar sensory evaluations.

The above-mentioned issues are evident in the sensory evaluation for various competitions and conformity evaluations of traditional vinegars, such as the two PDO (protected denomination of origin) Italian Traditional Balsamic Vinegars (ABT): ABT di Modena and ABT di Reggio Emilia [21]. On the surface, this does not seem to entail any problems since conformity evaluations and competitions take place at different times and places.

However, the overlap in the composition of the tasting panel usually leads to interferences in the results [22,23]. In the competition evaluation, the tasting panel must rank the vinegar samples, while in the conformity evaluation, the tasting panel only simply establishes whether the vinegar product complies with the relative quality standards. Due to the fact that the members of the panel for conformity and competition assessment often overlap, the impartiality of the sensory evaluation may be affected by the unclear distinction of the objectives of the ABT vinegar sensory evaluation in competition and conformity evaluations [23]. Therefore, clarifying evaluation methods and addressing cognitive and procedural issues are particularly important to ensure the objectivity and effectiveness of sensory evaluation for vinegar.

## 2. Importance of Vinegar Sensory Analysis to Find Potential Consumers

Sensory analysis is a very important and useful tool for the descriptive completeness of vinegar, guiding the production process and developing precise market strategies.

**Figure 1:**
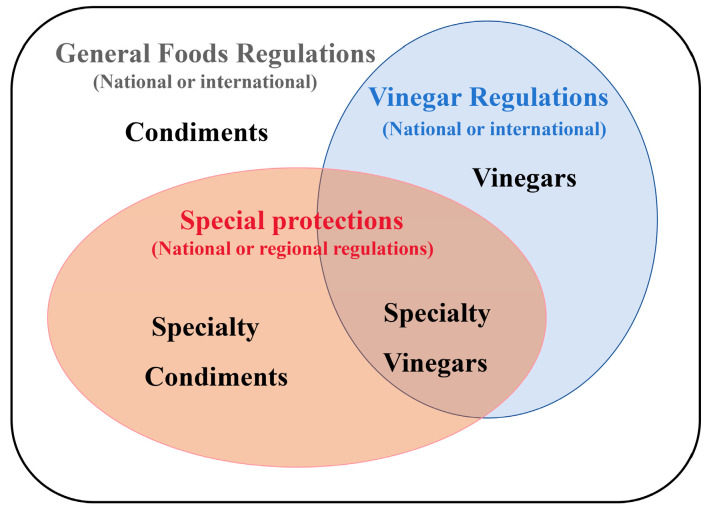
Different levels of legal status for Italian vinegars in the vinegar market (figure taken and modified from Corradini, 2018 [26]).

There are many products associated with vinegar in the marketplace. For example, in Italy, based on laws and regulations on vinegar and related products [24,25], besides the two PDO balsamic vinegars, there are various products that refer to balsamic vinegars or condiments on the market (Figure 1), which greatly complicates consumers’ choice.

Faced with such a wide range of vinegar and its relative products, consumer preferences are not only influenced by sensory attributes but also involve social, economic and cultural factors. Although it can be assumed that social, economic and cultural motivations are not important in driving consumer preference, the opposite is absolutely true. As far as Italian balsamic vinegar is concerned, at least two variables, personal taste and intended usage, influence the decision-making process. For personal taste, normally, certain sensory attributes such as the pungency, acidity, sweetness and viscosity of vinegar heavily affect the choice of a vinegar product. With regard to the intended usage, usually, viscous balsamic vinegar that does not flow very easily is suitable for decorating dishes, not only because of its taste, but also, it is nice to look at, whereas vinegar that flows easily with a strong pungent connotation is appreciated as a condiment for vegetable seasoning and pickled meat accessories.

In a nutshell, there are a very few people who like pungent and acidic balsamic vinegar, while many like low-pungent, low-sour and sweet vinegar.

Knowing the distribution of the consumer in terms of sensory preferences is highly valuable to develop market strategies for vinegar and its relative products, especially for the broad product category of generic balsamic condiments and ABT vinegars (Figure 1). What predominantly drives the purchase of ABT vinegars can be attributed to socio-economic and cultural aspects. Among them, their prestige and important communicative value are foremost. In the case of generic balsamic vinegars, they are subject only to food legislation and do not have special production specifications, so they do not require ageing and can be prepared by adding a wide range of ingredients, preservatives, thickeners and other food additives (Table 1). On the other hand, protected geographical indication (PGI) vinegars must meet the specifications of the relevant production specification, which limits the ingredients to only cooked must and/or concentrate and wine vinegar [27]. And for the preparation of ABT vinegar, a PDO product, only cooked must can be utilized (Table 1). In addition, it involves a long period of ageing in barrels, set up in series of five or more and managed [28].

Although it is very difficult to modify the sensory attributes of ABT vinegars, partly because ABT vinegar must comply with the specifications of the production specifications so that no ingredient is added other than cooked must [23,30], some technological tricks have been employed to modify the sensory characteristics of ABT vinegar. For instance, the viscosity of ABT vinegar can be increased by concentrating the cooked must or placing the barrels in rooms with low relative humidity [31,32]. In particular, widening the hole of the barrels has been proven to be a very effective method to enhance the viscosity of ABT vinegars. In fact, nowadays, all barrels have a larger open hole covered with a piece of cloth, whereas ancient barrels commonly possess smaller open hole and wooden stoppers [32,33]. Another sensory attribute of ABT vinegar, taste acidity, can also be modified by harvesting grapes early [34], as is carried out for sparkling wines [35]. However, it is a pity that ABT vinegar production specifications prescribe that grapes must be harvested when they are very ripe. The pungency of ABT vinegar, which is not an odour but the irritation of the nasal mucous membranes due to acetic acid, can be modified by guiding the fermentation process and closing the barrels, too [32,36].

The opposite case is represented by balsamic condiments that are not subject to controlled designations of their origins. A food technologist can design an ideal product for any use and for any consumer group, provided that its production is cost-effective. A food expert technologist can make viscous, sweet, sour and pungent balsamic seasonings in any ratio and combination because he or she can add some food ingredients and additives for any technological need. To sum up, in the market, balsamic condiments with low production costs are a powerful competitor to ABT vinegar. But are they really competitors of ABT vinegar? Yes and no: ‘yes’ is because balsamic condiments are appreciated by many consumers due to their lower prices; ‘no’ is because ABT vinegar is a prestigious product, and in many cases, it has a more important purpose than its food consumption since it is an exclusive, representative item and an important gift.

In reality, the real commercial competitors of ABT vinegar are the products that trick consumers into thinking that they are buying ABT vinegar, but actually, they are obtaining a normal condiment. There are many vinegar-like condiments on the market that correctly adhere to commercial legality, and they are difficult to distinguish from ABT vinegar if proper care is not taken during the purchase process. Some of them are produced and marketed by the same companies who also produce ABT vinegars.

## 3. The Shortcomings of the Existing Vinegar Sensory Analysis

### 3.1. The Shortcomings of the Tasting Procedure for Vinegar

For vinegar conformity, vinegar products must meet specific administrative requirements and pass the sensory judgement, both of which are carried out by independent certification agencies. And the sensory judgement is carried out by appropriately trained tasters who must be familiar with the criteria and specifications defined in the production specifications. In order to guarantee impartiality, the tasters are drawn from an appropriate group of vinegar product connoisseurs. Furthermore, the procedure of the sensory evaluation requires that the vinegar samples are unlabelled. To summarize, the tasting procedure for vinegar conformity is well structured to guarantee both the independence and impartiality of judgement.

However, some issues still exist in the vinegar conformity evaluation. First of all, there are issues with the sensory perception registration form because it is entirely analogous to the one used for the vinegar competition and employs a lexicon that does not comply with the indications of the ISO standards for tasting [23]. Furthermore, the sensory judgement of the vinegar conformity evaluation is usually made by a small and highly homogeneous group of product connoisseurs who have an ideal product model to guide their sensory perceptions [37].

The fact that sensory perceptions are recorded in the same manner for competition and conformity attributes, and the fact that the tasters are the same in the sensory panels of competition and conformity attributes, usually means that the tasting results for vinegar competition and conformity evaluations are strongly influenced by the unintentional predisposition of the panel members. It is evidenced that a high number of non-conformity vinegar is frequently observed for vinegar samples submitted for sensory evaluations of the conformity of vinegar. It is as if panel members have a very ideal model of vinegar, so vinegar samples that deviate from the model are penalized.

Conformity assessors and other tasting panel members are subjected to routine training and a timely assessment of their performance. This is well performed, reasonable and broadly satisfactory, but there are still some limitations. In the overall evaluation of vinegar, the reliability of the judge or taster is highly dependent on his or her ability to give results that are consistent with the panel’s average judgement and especially to give very similar evaluation results for an identical vinegar sample presented without identification. So, the most instinctive behaviour of tasters who know that they are being evaluated is to temper their perception close to the average value, and if he or she encounters vinegar that is unknown to him or her, he or she will simply say that it is good or bad.

### 3.2. The Shortcomings of Descriptors in Vinegar Sensory Analysis

The vocabulary currently used for vinegar descriptors is sometimes imprecise and redundant and leaves very wide margins of discretion. Some descriptive terms such as finesse, fullness or frankness are very generic and not measurable in terms of sensory attributes, meaning that the tasters are completely free to associate them with their own subjective sensory scale. Other descriptors such as acidity, with reference to olfaction, are inaccurate and disrespectful to the real perceivable sensation, which actually is pungency due to the trigeminal irritation of acetic acid.

In the sensory analysis of vinegar, the submitted vinegar samples must comply with the limits of refractometric response (Brix) and total acidity. The Brix value and density of a pure sugar solution can be converted from one to the other. But for vinegar, using a Brix value to calculate density is incorrect since vinegar is not a pure solution; there are many other solutes and at least two solvents: water and acetic acid. Even more so, it is incorrect to consider the Brix value as a measure of the sugars presented in vinegar. Unfortunately, this is what happens.

With regard to total acidity, it should be replaced by the more appropriate term titratable acidity, which mainly includes two types of acids, volatile acidity and fixed acidity. Volatile acidity is almost exclusively due to acetic acid in vinegar, while fixed acidity is mainly due to the organic acids from grape such as malic, tartaric and very little citric acid and sometimes gluconic acid [38,39]. The organic acids of grape are perceived in the mouth, while acetic acid plays a very important role during olfaction because it irritates the nasal mucous membranes and is perceived as pungency. The term pungency is the one that best expresses the olfactory sensation of vinegar in general, and it should replace the current olfactory descriptors of volatile acidity or acetic acid found in vinegar sensory analysis sheets.

Unfortunately, at present, tasting panels are provided with the Brix and total acidity values of a vinegar sample during the vinegar sensory analysis. This information significantly influences the subsequent sensory analysis because it is blatantly suggested that the Brix value is used to formulate the gustatory and visual evaluation of viscosity. These influences were the subject of studies some time ago [23].

## 4. Structuring a Vinegar Sensory Evaluation System: Key Descriptors, Recording Methods and Temporal Sequences

### 4.1. Important and/or Necessary Descriptors for Vinegar Evaluation

**Figure 2:**
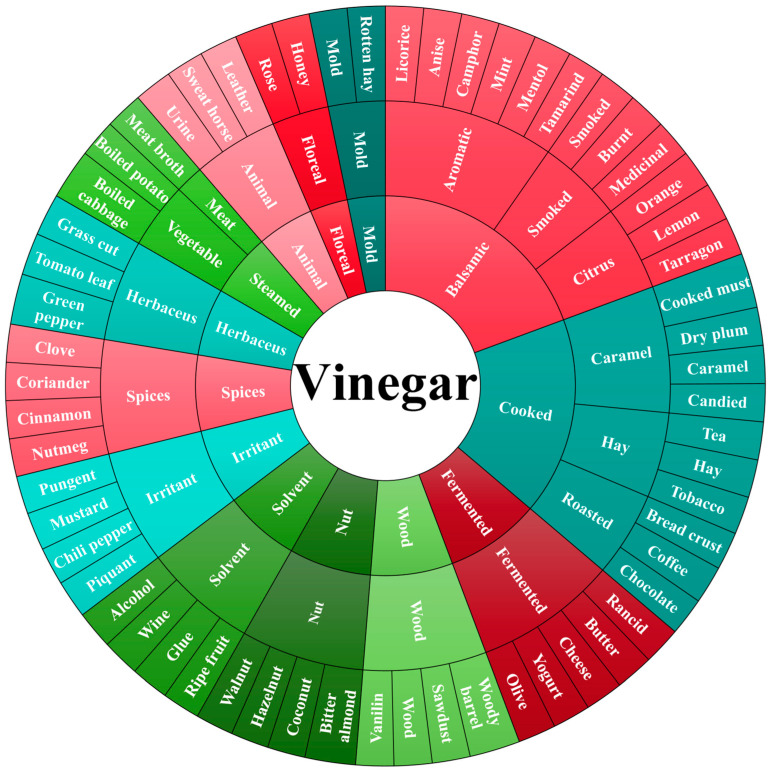
A sensory wheel of vinegar aromas (the aroma description of vinegar from the inner circle to the outer circle becomes increasingly specific and clear).

The perception record sheets, currently in use, adopt a vocabulary that is not always appropriate and does not conform to ISO indications [40]. The terms used for vinegar sensory descriptors must be clear, precise and shareable, allowing them to be easily traced back to experiences of common living and feeling. So, we develop a sensory wheel of vinegar aromas (Figure 2) in order to help consumers or tasters to visualize the different flavours, scents, and aromatic qualities [26]. Here, we only take the olfactory stimulation produced by vinegar as an example to explain: the stimulation olfaction of vinegar is a very particular sensation and common to all tasters and consumers by smell. So, calling it ‘volatile acidity’ or ‘acetic acid’ in the current perception description of vinegar is not right, and the correct term should be clear, precise and shareable, using ‘irritancy’ or ‘pungency’, which is more accurate and relatable.

The sensory descriptors of vinegar, as of any food product, can be a very long list. How many of them have a discriminating and/or characterizing weight in vinegar sensory evaluation is the question to be answered in order to prepare the appropriate sensory analysis procedure for vinegar. Obviously, the number of sensory descriptors should be as minimal as possible since each additional descriptor may add background noise. Let us imagine that we are attending a vinegar tasting test and are asked to evaluate 10 sensory descriptors for each vinegar sample. Obviously, this requirement may quickly lead to sensory fatigue and the impossibility of effectively evaluating more than two or three vinegar samples per tasting session. Certainly, some descriptors hold clear hierarchical importance, including clarity, colour, pungency, acidity, sweetness and viscosity.

### 4.2. The Recording Board for Vinegar Sensory Perception

The perception record sheet is a basic tool of the vinegar sensory analysis, which includes several aspects [23]. Here, we will only dwell on the necessity of large scales of scoring on the record sheets in sensory judgments for vinegar competitions since it can facilitate the selection process by significantly decreasing the percentage of vinegar samples with identical scores. However, in the conformity assessment for vinegar, identical scores are irrelevant since the vinegar samples are simply categorized as either compliant or non-compliant with the specified quality standards.

### 4.3. Temporal Sequence of Vinegar Sensory Perceptions

In the current vinegar procedure, a visual examination is often the first sensory evaluation, which has a significant influence on subsequent perceptions since tasters tend to attribute high olfactory and gustatory scores to vinegar samples that are evaluated positively in the visual examination. Even if the scores for the visual descriptors such as viscosity, colour and clarity take a minimal or even zero weight coefficient, the scores from the olfactory and gustatory attributes are strongly affected by the visual scores of the vinegar samples [41].

## 5. The Practice of the Sensory Evaluation of Vinegar: Taking the Italian Traditional Balsamic Vinegar as an Example

### 5.1. The Factors Affecting Vinegar Sensory Evaluation

Generally speaking, the sensory evaluation for Italian ABT vinegar is a structured and scientific process that covers the professional composition of the tasting panel, systematic evaluation structure, reasonable questionnaire design and strict tasting procedure [21,23]. But some issues still exist. For instance, the order of sensory evaluation is very important in the ABT vinegar tasting procedure, which was realized a long time ago by people who drafted the current procedure [23]. In particular, they had observed that the visual examination had a great influence on the final judgement, and the corrective action was to limit its scoring. But this does not solve the problem because the initially conducted visual assessment can affect the following sensory perceptions. So, for the vinegar sensory analysis, we proposed very early on to carry out the visual analysis as a last resort [23].

Beyond the procedural order, taster judgments are also heavily influenced by environmental factors, including shared tables, crowded rooms, and the presence of very experienced tasters or those considered as such, who can heavily influence the free sensory expression of tasters, especially less experienced or more dubious tasters [42]. Another critical factor is the psychological sensitivity of tasters to the evaluation of their sensory performance, which strongly conditions the taster’s freedom of judgement by making the taster refrain from expressing extreme scores, even in the case of very precise and strong perceptions. In short, to be on the safe side, the taster adjusts his or her evaluation towards a hypothetical average value. In this regard, it is sufficient that eliminating extreme scores when calculating the average is still in force.

### 5.2. Taster Training

The training and evaluation of tasters are very particular and require attention at the highest level. A taster who unconsciously feels under scrutiny will modify his or her sensory judgement and eliminate the more extreme values and express his or her perceptions using values close to the average one.

### 5.3. Information on Vinegar Samples

Accompanying the samples to be evaluated with information on their composition is the best way to invalidate the subsequent sensory analysis since the taster is required to modify his or her perception in relation to the value of the analytical data, which is a very serious error because it is based on the erroneous assumption that the analytical and sensory data are correlated. This assumption may be true for some sensory active compounds in a pure solution and within precise concentration limits, but it is not at all true in complex mixtures with sensory antagonistic compounds, as is the case with vinegar. To simplify this, if we taste a sugar solution and taste it again after the addition of a few drops of lemon, it will seem less sweet despite having the exact same sugar concentration. The information accompanying the vinegar samples significantly affects their sensory expression and limits the taster’s independence of judgement, especially when the Brix values of vinegars are used as a calibration parameter for perceived viscosity [43].

### 5.4. Other Information of Vinegar Samples

In addition to the information described above, in the tasting session, the vinegar samples must be distributed in known quantities (e.g., 3 mL) and in a darkened container to prevent the colour and viscosity of the vinegar influencing the judgement of the other sensory attributes. The visual evaluation of colour and viscosity must be performed only at the end of the sensory analysis. The tasting environment must be free of noise and adequately lit to minimize the impact of external interference on sensory perception. Questionnaire design and data recording are important for sensory evaluation. The questionnaire adopts a unipolar or bipolar interval scale to record the sensory data quantitatively. Descriptors need to be clear, precise, and not redundant to avoid overly similar terms such as ‘smell freshness’ and ‘smell fineness’ to improve assessment efficiency and reduce confusion.

### 5.5. The Practice of the Sensory Evaluation of the Italian Traditional Balsamic Vinegar

We started the sensory analysis for Italian ABT vinegars at the beginning of this century. Below is a summary of the significant results and the procedure adopted. The sensory analysis method for Italian ABT vinegar established in 2009 has been followed in more recent years by an extensive sensory analysis of a number of vinegar samples of different origins and provenances to obtain an idea of the great variability of vinegar produced in the world [44].

In the sensory procedure for Italian ABT vinegar used in 2009, the indications of the ISO standards were slavishly followed; meanwhile, in order to enrich the descriptive vocabulary of the sensory attributes of vinegar, a flexible procedure was followed to make maximum use of the sensory capabilities of the tasters. Specifically, in the first sensory analysis, 25 samples of ABT vinegar were selected, including some vinegars that were not aged for long, which were strictly speaking not ABT vinegars, to facilitate the development of a sensory lexicon as representative as possible for the real quality of the available vinegars. All vinegar samples were coded with a randomized alphanumeric code and served in small tasting cups one at a time and with a waiting interval for the tasters to recover their full performance. The tasting panel consisted of 21 non-professional tasters, who were briefed on the basic guidelines required to accurately describing the ‘flavour’ and ‘aroma’ characteristics following Bérodier et al., 1997 [45], and the ‘texture’ characteristics according to Lavanchy et al., 1993 [46]. The members of the panel were selected from a diverse group in terms of cultural backgrounds, eating habits, ages and genders. The absence of professional tasters and ABT vinegar connoisseurs may seem unusual to someone, but this was a deliberate choice to avoid the cognitive biases and preconceived notions that might influence and skew sensory judgement.

The variety of vinegar samples, combined with the judicious composition of the panel, proved effective in generating a coherent list of terms for identifying the most representative descriptors of the sensory profiles of the vinegar (Table 2). And in order to avoid any possible conditioning, the tasters were not given any information about the vinegar samples, such as ages, producers, compositions or configurations of the vinegar origin. For each tasted vinegar sample, a list of attributes freely and subjectively assigned by each taster was recorded. Subsequently, a moderator, acting as the ‘panel head’, initiated discussions with each taster and the entire panel to ensure a shared understanding of and alignment with the recorded terms. After all the tasters had reached an agreement, a careful analysis of the literature data continued in order to identify the terms to be used as possible reference standards for the attributes recognized in the ABT vinegars (Table 3).

It is worth pointing out that not all of the descriptors identified by the tasters were specific to aged ABT vinegar as some of the samples were their intermediates, such as vinegars not aged for very long or fermented cooked musts. On the other hand, the descriptors including prune, liquorice, chocolate, coffee, carob and spices were only associated with the aged ABT vinegar samples, as if they were the result of the long ageing process. Another interesting observation can be deduced from the perceived sensation for the taste descriptors: in all aged vinegars, acid, sweet, bitter and salty were present with varying degrees of intensity. Additionally, for the trigeminal sensation of astringency, terms such as ‘pungent’ and ‘spicy’ were added to the taste descriptors (Table 3).

Combining our practice of the sensory evaluation of vinegar with the sensory description of Italian balsamic vinegars from the literature [23], we can categorize the Italian ABT vinegars into the following groups: (1) very aged ABT vinegar with high pungency and low viscosity and/or little sweetness, which is liked by very few consumers and tasters; (2) equally genuine and old ABT vinegar with low pungency, high viscosity and sweetness, which is liked by very many consumers and rated well upon tasting; and (3) balsamic vinegar with little pungency and a very balanced sour/sweet taste, the enjoyment of which is very random.

## 6. Discussion

Sensory analysis can serve diverse purposes for food, and thus, the methodology must be customized for specific goals [4,51]. In the case of vinegar, sensory evaluation differs significantly depending on the purpose: determining whether the vinegar products meet the relative standard or in competition merit rankings. It can be determined that since the two purposes are different, the sensory analysis procedures must also be appropriately different from both objectives, starting with the sensory perception acquisition sheet, the use of reference standards, and the number and type of descriptors to be used. For example, the wide scale of scoring values on the scorecards currently in use is appropriate and necessary in the sensory analysis of vinegar competitions but completely useless, if not harmful, in vinegar conformity judgments [52]. The broad score scale is very useful in vinegar competitions because it significantly decreases the number of samples with identical scores; otherwise, repeated reassessments would be required [23]. In contrast, in vinegar conformity evaluations, the broad scale would make the scorecards too similar to those used in vinegar competition evaluations.

The sensory procedure can make use of standard samples to precisely define the limits of acceptability and categories of the vinegars submitted for sensory evaluation in the attribution of conformity [53]. In particular, reference samples favour the consistent evaluation of samples under examination during tasting sessions by different panels and limit sensory judgement that deviate from the pre-set quality standards [54].

Vinegar rankings are certainly favoured by registration forms that provide ample scoring, such as those currently in use. For example, in recent times, a viscous and not very pungent type of Italian balsamic vinegar has gradually established itself, and the more flowing and pungent one has almost disappeared [43,55]. One of the reasons for this trend is that tasters and consumers associate viscosity with quality and believe that vinegar with higher viscosity has better quality, meaning that viscous Italian balsamic vinegar is perceived as qualitatively better than flowing ones. In vinegar competition evaluations, the very difficult task is to evaluate vinegars for their genuineness and sensory complexity regardless of their viscosity [16].

In short, sensory analysis can be used for different purposes, and for each, a specific procedure is required, starting with the method of detecting sensory perceptions.

In the future, studies should further explore the relationship between sensory assessment and consumer preferences for different vinegars from different countries or regions and how to develop more scientific and impartial sensory assessment standards and methodologies to enhance the reliability and practicability of vinegar assessments.

## Figures and Tables

**Table 1:** Vinegar and its relative products associated with the term balsamic in Italy *.

Product Categories	Main Ingredients	Permitted Additives					Ageing Time
		Dyes	Aromas	Thickeners	Emulsifiers	Preservatives	
Traditional Balsamic Vinegar PDO **	Cooked must	No	No	No	No	No	At least 12 years
Balsamic Vinegar of Modena PGI	Wine vinegar, concentrated and/or cooked must	Caramel	Caramel	No	No	Sulphites ***	At least 60 days
Glazes, Sauces and Balsamic Condiments	Wine vinegar, sugars	Yes	Yes	Yes	Yes	Yes	No

* From Giudici, 2023 [29], ** both Modena and Reggio Emilia PDOs, *** presence due to residues from grape and must processing.

**Table 2:** Sensory attributes used to describe Italian ABT vinegars *.

Aspect	Aroma	Taste	Texture	Trigeminal Sensation
Colour	Caramel	Acid	Viscosity	Astringent
	Wine/Cooked must	Sweet		Pungent
	Wood	Amaro		Spicy
	Fruit	Salty		
	Plum/prune			
	Acetic acid			
	Honey			
	Apple			
	Liquorice			
	Mustard			
	Vanilla			
	Carob			
	Spices			
	Coffee			
	Chocolate			

* From Giudici, 2023 [29].

**Table 3:** Sensory attributes and reference standards used for the evaluation of ABT vinegars.

Attributes	Descriptions	Standards	References
Caramel	Aroma and flavour associated with caramel syrup	Ildia-type caramel syrup	
Wine/ cooked must			
Wood			
Fruit	Aroma associated with different fruits		
Prune		Mixture of 1–2 mL plum juice and 25 mL red wine	Noble et al., 1987 [47]
Acetic acid		Mixture of 2 drops of glacial acetic acid and 50 mL red wine	
Honey		Mixture of 5–8 mL honey in white wine	
Apple		Fresh apple slices in 5 mL apple juice and 25 mL white wine	
Liquorice		Mixture of 1 drop of aniseed extract and 50 mL red wine	
Mostarda			
Vanilla	Aroma associated with vanilla extract	Mixture of 1–2 drops of vanilla extract in 25 mL red wine	
Carob			
Spices		2–3 ground white pepper granules in 25 mL red wine	
Coffee	Aroma associated with coffee beans	2–4 ground coffee granules	
Chocolate	Aroma associated with chocolate	2–5 mL chocolate flavouring or ½ teaspoon cocoa powder in 25 mL red wine	
Acid	Sensation produced by aqueous citric acid solution	Aqueous solution of citric acid (0.43 g/L)	Giudici et al., 2009 [41]
Sweet	Sensation produced by aqueous sucrose solution	Aqueous sucrose solution (5.76 g/L)	
Bitter	Sensation produced by aqueous solution of various compounds including caffeine	Aqueous caffeine solution (0.195 g/L)	
Salty	Sensation produced by aqueous solution of sodium chloride	Sodium chloride aqueous solution (1.19 g/L)	
Viscosity	Instrumental measurement of slip resistance in rotary motion at controlled speed	Calculated as stress/speed ratio under Newtonian conditions	Falcone et al., 2007 [48]
Astringent	Attribute associated with the sensation of astringency produced by pure substance or specific mixtures	Aqueous solution of potassium aluminium sulphate hydrate (0.05% aluminium by weight)	Jellinek [49]
Pungent	Irritating sensation perceived inside the mouth	1.5 mL of 0.5% by weight aqueous solution of Cayenne after 5 min boiling and subsequent filtration with 10 g of quark	Prescott and Stevenson [50]
Spicy	Sensation of heat produced inside the oral cavity (e.g., by peppers)	Olive oil	Jellinek [49]

## Data Availability

No new data were created or analyzed in this study. Data sharing is not applicable to this article.

## Abbreviations

The following abbreviations are used in this manuscript:

ABT	Italian Traditional Balsamic Vinegar
PDO	Protected designation of origin
PGI	Protected geographical indication
